# Supplementary material for: Splice-Junction-Based Mapping of Alternative Isoforms in the Human Proteome
Source: Cell Rep. Author manuscript; Available in PMC 2020 Jan 15. (PMC6961840; doi:10.1016/j.celrep.2019.11.026)
Supplement: 2 [file NIHMS1546469-supplement-2.zip › DF1/PXD010154/Testis/Testis_19_ATXN3_EEGSLCAQHCLNNLLQGEYFSPVELSSIAHQLDEEER.pdf]

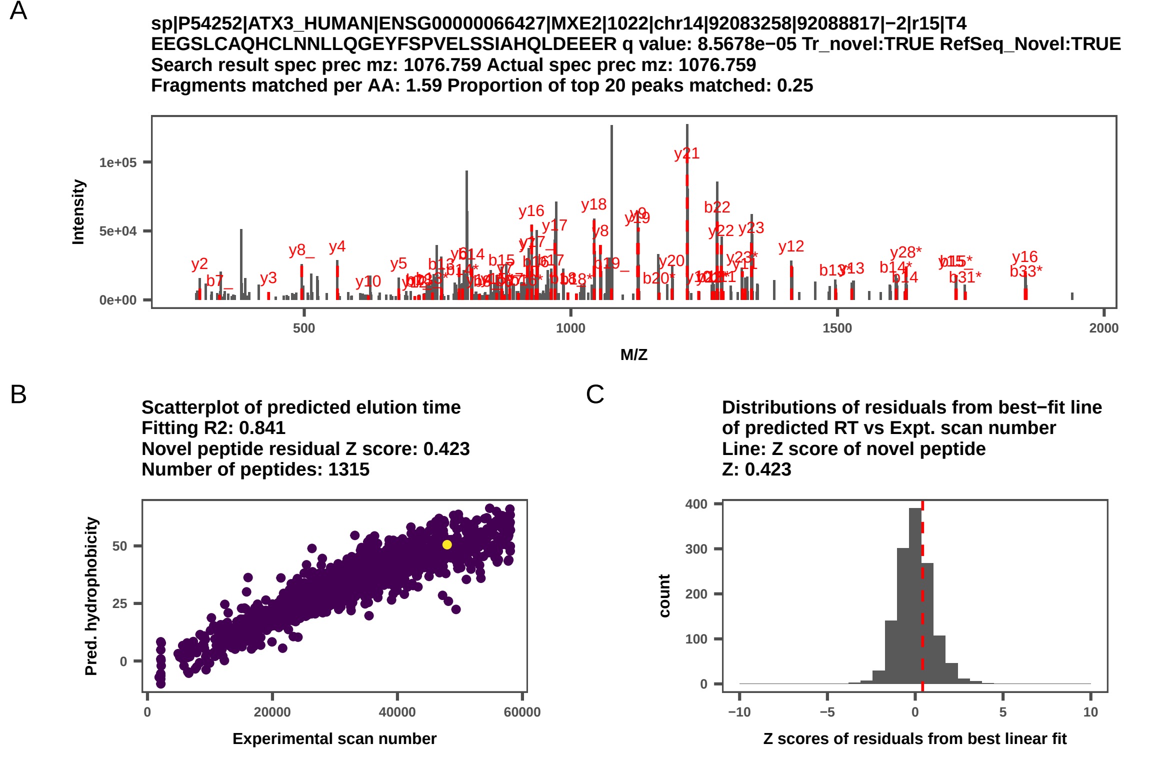
A
sp|P54252|ATX3_HUMAN|ENSG00000066427|MXE2|1022|chr14|92083258|92088817|−2|r15|T4
EEGSLCAQHCLNNLLQGEYFSPVELSSIAHQLDEEER q value: 8.5678e−05 Tr_novel:TRUE RefSeq_Novel:TRUE
Search result spec prec mz: 1076.759 Actual spec prec mz: 1076.759
Fragments matched per AA: 1.59 Proportion of top 20 peaks matched: 0.25
Intensity
1e+05
5e+04
0e+00
y2
y3
y4
y5
y10
y16
y17
y18
y8
y21
b22
y23
y12
y20
b15
y16
b33*
M/Z
500
1000
1500
2000
B
Scatterplot of predicted elution time
Fitting R2: 0.841
Novel peptide residual Z score: 0.423
Number of peptides: 1315
Pred. hydrophobicity
50
25
0
0
20000
40000
60000
Experimental scan number
C
Distributions of residuals from best−fit line
of predicted RT vs Expt. scan number
Line: Z score of novel peptide
Z: 0.423
count
400
300
200
100
0
−10
−5
0
5
10
Z scores of residuals from best linear fit
